# Ankle sprain history and clinical outcome have limited influence on walking and running biomechanics among runners: a cross-sectional study

**DOI:** 10.3389/fspor.2025.1553995

**Published:** 2025-09-08

**Authors:** Jean-Louis Peters-Dickie, Christine Detrembleur, Margaux Bertrand, Emma Detrembleur, Anh Phong Nguyen

**Affiliations:** ^1^ Neuro Musculo Skeletal Lab (NMSK), Institut de Recherche Expérimentale et Clinique, Secteur des Sciences de la Santé, Université Catholique de Louvain, Brussels, Belgium; ^2^ Musculoskeletal Rehabilitation Research Group, Department of Rehabilitation Sciences, Katholieke Universiteit Leuven, Bruges, Belgium; ^3^ Clinical Motion Analysis Laboratorium (CMAL), Universitaire Ziekenhuizen KU Leuven, Lubbeek, Belgium; ^4^ Faculté des Sciences de la Motricité, Université Catholique de Louvain, Ottignies-Louvain-la-Neuve, Belgium; ^5^ The Running Clinic, Lac Beauport, QC, Canada

**Keywords:** chronic ankle instability, coper, locomotion, gait, biomechanical phenomena, ground reaction force, perceived instability, self-reported function

## Abstract

**Background:**

Lateral ankle sprain (LAS) is prevalent among runners, with many developing chronic ankle instability (CAI). While CAI is associated with many motor-behavioral, sensory-perceptual, and pathomechanical factors, its impact on gait biomechanics remains unclear. This cross-sectional study aimed to assess gait biomechanics and other factors contributing to CAI in runners.

**Methods:**

Seventy participants (47 men and 23 women) were categorized as healthy (*n* = 24), acute LAS (*n* = 17), CAI (*n* = 16) and copers (*n* = 13). Walking and running spatiotemporal, kinetic and kinematic parameters were collected on an instrumented treadmill. Rehabilitation-oriented assessment outcomes were also assessed. One-way ANOVA or Kruskal–Wallis tests were used, along with their corresponding post-hoc tests. Effect sizes (g or r according to normality) were reported.

**Results:**

Runners with CAI and acute LAS reported significantly greater perceived instability (*r* = 0.68–0.86) and worse self-reported function (*r* = 0.47–0.67) than healthy controls and copers. However, running biomechanics did not differ between groups, suggesting that traditional biomechanical assessments at comfortable speeds may not be sensitive to functional deficits in CAI. A notable finding was the lower mechanical work recovery during walking in copers compared to healthy controls (g = 0.98).

**Conclusion:**

These results highlight the importance of considering self-reported function and perceived instability when assessing LAS and CAI. The absence of gross running gait alterations suggests that rehabilitation could safely integrate running early in recovery. However, more demanding tasks or advanced biomechanical modeling techniques may be needed to identify residual gait impairments.

## 1 Introduction

Running is one of the most popular sports in the world. The practice of this sport is associated with high rates of running related injuries, with estimated prevalences of 7.7–17.8 injuries per 1,000 h of running (1). Ankle sprain, and more specifically the lateral ankle sprain (LAS), is the most common traumatic running-related injury (2), with incidences varying from 10% among recreational runners to 21% among elite runners (3).

Approximately 40% of people undergoing a first LAS will experience recurrent ankle sprains or episodes of giving ways, along with persistent symptoms including pain and perceived instability, which are the recognized criteria for chronic ankle instability (CAI) (4). The symptoms and altered function caused by CAI (5) thus particularly threaten sport participation and performance in runners. Post-traumatic osteoarthritis is also frequent following LAS and could require expensive and non-functional ankle replacement surgeries (6). The patients who recover to pre-injury levels of physical function without evidence of CAI one year after their last LAS are referred to as “copers” (7).

The current model explaining the occurrence of CAI following an initial LAS identifies three primary categories of impairments: motor-behavioral, sensory-perceptual, and pathomechanical. Altered walking and running gait biomechanics are part of the motor-behavioral impairments that contribute to CAI (5). More specifically, the walking and running kinematics of individuals with CAI were characterized by a laterally deviated center of pressure trajectory compared to control participants. During walking, there was also an increased shank external rotation and ankle joint inversion, and a decreased ankle joint dorsiflexion. Similarly, the running of individuals with CAI was associated with increased shank external rotation and ankle inversion, as well as a more plantarflexed ankle position, compared to control subjects (8). A few studies investigating running biomechanics in CAI have included copers or individuals with a recent LAS (9–11). Such comparisons are essential because these clinical groups experienced a similar initial injury (7), enabling researchers to formulate hypotheses about why some individuals developed chronic symptoms while others did not. Additionally, despite the high prevalence of LAS in runners (3), no previous study has compared locomotion biomechanics among runners with varying clinical outcomes after LAS.

When suffering from LAS or CAI, it is recommended that patients pursue rehabilitation. However, many injured individuals do not seek treatment (12), and for those who do, the rehabilitation provided is often insufficient or improperly administered (6, 13, 14). Because LAS and CAI are related to various deficits (5), a recent consensus statement outlined the clinically relevant outcomes that should be assessed in every patient with a LAS (15). Rehabilitation can then be tailored to address the identified impairments, a strategy referred to as ‘rehabilitation-oriented assessment’ (ROAST). This assessment comprises pain, swelling, range of motion, arthrokinematics, ankle strength, static and dynamic balance, evaluation of gait, level of physical activity and patient-reported outcomes measures of perceived instability and self-reported function (15). Many of these outcomes are part of the theoretical model of CAI (5), and some such as balance are risk factors for LAS (16, 17). No previous study investigated the outcomes of the ROAST among runners with and without CAI. Consequently, rehabilitation for runners with a history of ankle sprain is likely suboptimal due to limited understanding of their clinical characteristics.

The primary objective of this study was to compare the walking and running biomechanics of participants with CAI to those of copers, healthy controls, and individuals who have recently sustained a LAS. We hypothesized that runners with CAI and acute LAS will exhibit altered walking and running biomechanics compared to healthy controls and copers. Besides, we expected copers to show slight alterations compared to controls. The secondary objective was to compare the outcomes of the ROAST among these four groups of participants. We hypothesized important between group differences regarding perceived instability and function, along with balance deficits.

## 2 Methods

### 2.1 Study design

This cross-sectional, comparative study followed the STROBE recommendations (18). STROBE checklist is available as Supplementary Material. During the recruitment, all participants signed an informed consent form. The procedure for this study complies with the local ethical committee (Comité d'éthique hospitalo-facultaire, approval number BE403201523492).

### 2.2 Participants

Active runners were recruited, defined as at least 6 months of consistent running experience and being able to run for an hour without stopping. Eligible participants were enrolled and assigned to one of the four study groups according to their LAS history and evidence of perceived instability according to the Cumberland Ankle Instability Tool (CAIT). Individuals who had no history of ankle sprain and no evidence of perceived instability (CAIT > 24) were included as healthy controls. The participants of the three other groups had to report at least one substantial LAS that required at least one day of immobilization or discharge of the ankle (4). Participants who had their last substantial LAS between 2 weeks and one year prior to study participation were included in the LAS group. Individuals who had their last substantial LAS more than one year ago were divided according to their CAIT scores: CAIT ≤ 24 for CAI and CAIT > 24 for copers (5). The participants included in the healthy control and coper groups had also to report excellent self-reported function. Therefore, they filled the Foot and Ankle Ability Measure questionnaire, and a cutoff score of 95% was used (5). A history of surgery on the lower limb or lower back, cardiovascular illness, neurological disease, degenerative condition, current pregnancy, and being younger than 18 years old were all exclusion criteria.

We used the French versions of the CAIT and FAAM in this study (19, 20), as French was the native language of our participants. The selection of these tools aligns with recent recommendations that patient-reported outcome measures for CAI should be evaluated based on their relevance to CAI populations, including aspects such as recalibration, detectability, and identifiability (21). The CAIT is a valid and reliable instrument with known minimal detectable changes and validated French adaptation (20, 22, 23). Similarly, the FAAM is valid, reliable, responsive to functional change, and available in a validated French version (19, 24).

### 2.3 Testing procedure

Following the completion of the questionnaires, participants had an individual appointment at the Neuromusculoskeletal laboratory in Brussels. Data collection took place from July to September 2023. Each participant received a ROAST clinical assessment by one investigator (E.D.), as well as a running biomechanics assessment by another investigator (M.B.). An information and consent letter was signed by every participant prior to any assessment.

The anamnesis was used to collect current pain intensity (11-point verbal numeric rating scale) and level of physical activity (Tegner activity level scale) (15). The physical examination first included ankle swelling using the figure of eight test with a one-quarter-inch wide measuring tape (25). Then, participants performed the Weight Bearing Lunge test. They were instructed to place their foot as far from the wall as possible while keeping the heel down and touching the wall with their knee. The examiner visually confirmed that the task was completed correctly. The participants performed three trials, and the mean distance between the hallux and the wall from the three trials quantified ankle dorsiflexion range (26). The Posterior Talar Glide test was used to quantify talus arthrokinematics. The participant therefore sat on the table, his/her ankle was placed into subtalar neutral position and the examiner gently pushed posteriorly the talus and the ankle into dorsiflexion until a firm capsular end (27). The angle between the lower leg and the vertical was measured using the “Clinometer®” app (App Store, © 2020 Phoenix Solutions) on a smartphone, which was placed against the tibia (28). The Balance Error Scoring System was performed. Participants were asked to hold balance during 20 s with eyes open and then 20 s with eyes closed, first on a firm surface and then on foam surface (Airex® balance pad, Airex AG®, Sins, Switzerland). They were asked to maintain balance during double leg stance, tandem stance, and single leg stance. Errors were counted for the following: hands off the hips, eyes open during an “eyes closed” condition, to take a step, to fall, perform a hip abduction or flexion of more than 30°, and lift the forefoot or heel off the ground. The maximum number of errors per condition was 10 (29). The modified star excursion balance test (mSEBT) was performed three times in each direction. A “Y” was drawn on the ground, and a tape measure was used to quantify the distance reached in the anterior, posteromedial, and posterolateral directions. Participants were instructed to reach as far as possible with one foot, lightly touching the ground, while keeping the other foot in contact with the floor. The mean distance per direction was normalized to leg length (30).

**Figure 1:**
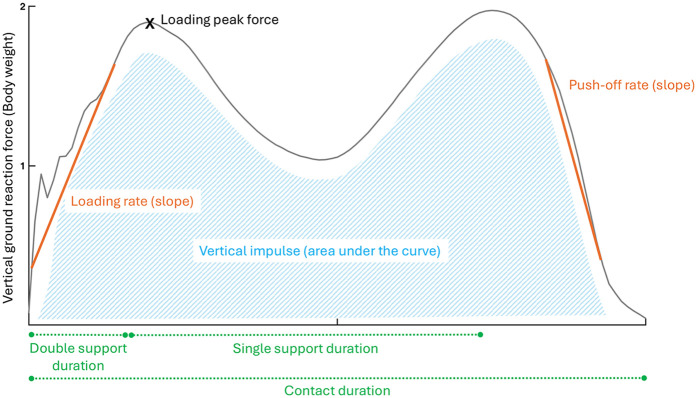
Walking biomechanical outcomes related to the vertical ground reaction forces. Orange (outcomes based on the slope), Green (spatiotemporal outcomes), Blue (outcomes based on the area under the curve), X (point of interest).

**Figure 2:**
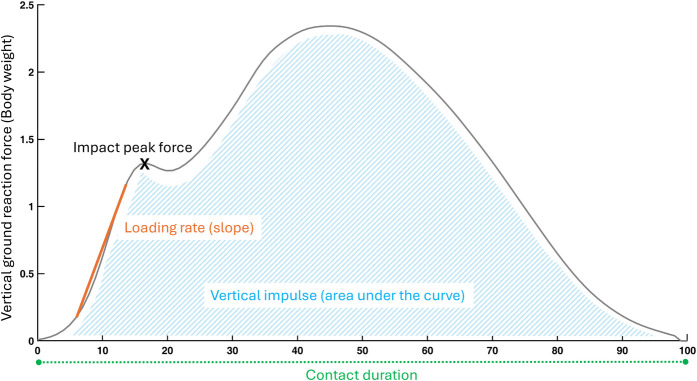
Running biomechanical outcomes related to the vertical ground reaction forces. Orange (outcomes based on the slope), Green (spatiotemporal outcomes), Blue (outcomes based on the area under the curve), X (point of interest).

Participants were also assessed on an instrumented treadmill while wearing their usual running shoes. They first walked for 3 min at a self-selected speed that matched their day-to-day walking speed. Participants ultimately ran for 5 min at a self-selected speed that would allow them to run for one hour while being able to converse. This intensity corresponded to 3 out of 10 on the category ratio (CR10) rating of perceived exertion scale (31, 32). The CR10 scale, administered verbally, defines 0 as “rest” and 10 as “maximal exertion” (33). During the last 20 s of running, the ground reaction forces were recorded at a frequency of 1,000 Hz by force sensors placed at the corners of the treadmill, and filtered using an 8th order Bessel low pass filter at 25 Hz with zero lag. This means that at running data collection, participants had spent 8 min on the treadmill, which was reported to be sufficient for an individual to stabilize running stride (34). The calculated spatiotemporal outcomes included cadence (steps per minute), stride duration (ms), step, contact and aerial durations (% of stride), and duty factor. Kinematic outcomes included support width (cm), vertical displacement of the center of mass (mm), path of the center of mass (mm), and step length (cm). Kinetic outcomes included total, vertical and antero-posterior mechanical work (J.kg^−1^.m^−1^), vertical, breaking and propulsive impulses (%BW.s), loading rate (%BW.ms^−1^), impact, active and breaking peak forces (%BW), and leg vertical stiffnesses (%BW.m^−1^). The details of data calculation are available in Supplementary Materials (35). Figures 1, 2 offer a visual representation of the outcomes related to the vertical ground reaction forces during walking and running.

### 2.4 Statistical analysis

For data with one value per side (left and right), a single side was included in the analyses. For CAI, copers and LAS groups, the leg to be analyzed was chosen as follows: (1) if the participant sustained a single or multiple LAS at a single side, that side was selected, (2) if the participant sustained LAS at both ankles, the most affected ankle according to CAIT questionnaire was selected, and (3) if CAIT were equal between sides, the right leg was selected according to biomechanical convention. For the healthy control group, the right leg was also analyzed.

Statistical analyses were conducted on SPSS software (version 27, IBM, Chicago, IL, USA), with a significance level set at *p* = 0.05 for all analyses, except *post-hoc* tests. First, discrete quantitative data (gender distribution) were compared between groups using a chi-squared test. Second, continuous quantitative data were assessed for normality within each group employing the Shapiro–Wilk test. Normally distributed outcomes were compared between groups using one-way analyses of variance (ANOVA). Subsequently, *post-hoc* multiple t-test comparisons were conducted with Bonferroni correction (*p* = 0.05/6), and Hedge's g effect sizes were calculated and interpreted as follows: <0.2 very small, <0.5 small, <0.8 medium, <1.2 large, <2 very large, and ≥2 huge (36). The Kruskal Wallis test was used as a non-parametric alternative, followed by Mann–Whitney *U post-hoc* tests with Bonferroni correction. The r effect size of the Mann–Whitney test was calculated by dividing the z statistic by the square root of the sample size, and the results were interpreted as follows: <0.3 small, <0.5 medium, ≥0.5 large (37). Eta squared (*η*²) and the 95% confidence intervals (CI) for *η*² were calculated for both the ANOVA and Kruskal–Wallis tests to represent the percentage of variance in the dependent variable explained by the independent variable. The CI for *η*² related to the Kruskal–Wallis were calculated using Bootstrapping with 1,000 iterations.

## 3 Results

### 3.1 Participants

**Figure 3:**
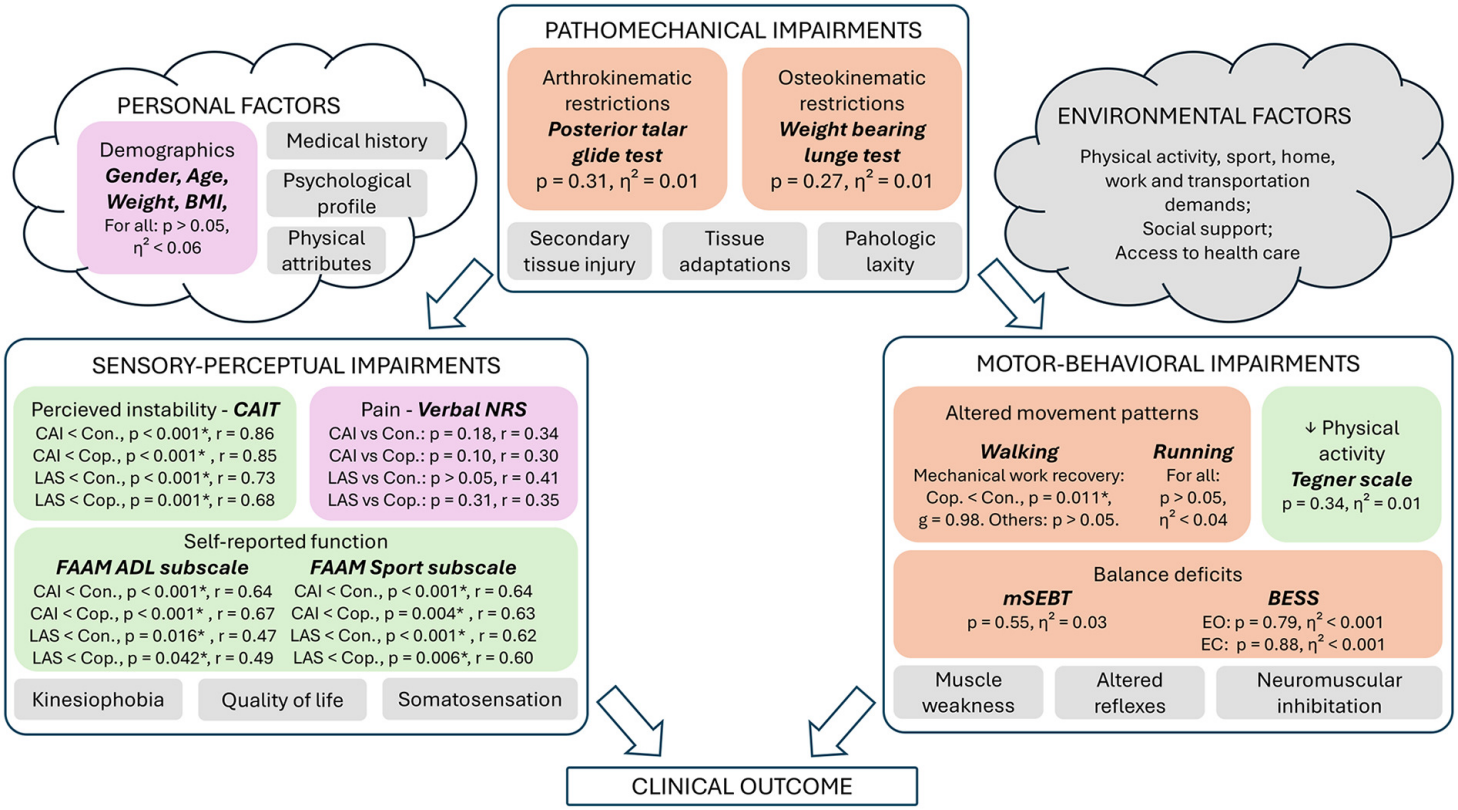
Results related to the clinical and biomechanical examinations. This figure depicts our results regarding the rehabilitation-oriented assessment (ROAST) outcomes (15), and was adapted from the model of Hertel and Corbett (5). Colors: Purple (anamnesis), Green (questionnaires), Orange (Physical examination), Grey (not assessed in this study). ADL, activities of daily life; BESS, balance error scoring system; CAI, chronic ankle instability; CAIT, cumberland ankle instability tool; Con., control group; Cop., coper group; EC, eyes closed; EO, eyes open; FAAM, foot and ankle ability measure; g, Hedge's g effect size related to the *post-hoc* t-test; LAS, lateral ankle sprain group; mSEBT, modified-star excursion balance test; NRS, numeric rating scale; p, *p*-value; r, effect size related to the Mann–Whitney *post-hoc* test; *η*², eta squared effect size related to the ANOVA and Kruskal–Wallis tests. *(statistically significant at *p* < 0.05).

Demographics and results of the ROAST are available in Table 1, as well as in Figure 3.

**Table 1:** Demographics and results of the rehabilitation-oriented assessment (ROAST).

Outcomes	Healthy controls (*n* = 24)		Recent LAS (*n* = 17)		CAI (*n* = 16)		Copers (*n* = 13)		*p*-value	*η*² effect size (95% CI)
	Mean	SD	Mean	SD	Mean	SD	Mean	SD		
Sex (M/F)^‡^	16 M/8 F		12 M/5 F		12 M/4 F		7M/6F		*p* = 0.66	/
Age (years)^‡^	27.92	8.65	24.76	5.56	30.94	11.37	27.31	5.27	*p* = 0.14	0.04 (0.02; 0.05)
Weight (kg)^†^	73.13	8.87	75.38	14.19	76.73	13.44	68.44	7.50	*p* = 0.23	0.06 (0; 0.17)
Height (cm)^‡^	178.21	7.65	178.47	9.31	178.63	9.70	173.85	7.03	*p* = 0.23	0.02 (0; 0.03)
BMI (kg.m^−2^)^‡^	23.05	2.80	23.50	2.95	23.99	3.46	22.62	1.83	*p* = 0.73	<0.001 (undefined)
CAIT (/30)^‡^	28.92	1.56	20.88	6.30	21.13	2.70	28.38	1.85	*p* < 0.001*	0.80 (0.76; 0.80)
FAAM_ADL (%)^‡^	99.71	0.91	95.53	6.45	97.31	2.36	99.92	0.28	*p* < 0.001*	0.31 (0.29; 0.32)
FAAM_Sport (%)^‡^	99.38	1.53	81.53	24.07	92.69	8.51	99.54	1.13	*p* < 0.001*	0.38 (0.36; 0.39)
Tegner activity scale (/10)^‡^	5.71	0.75	6.18	1.51	5.94	1.12	5.38	0.96	*p* = 0.34	0.01 (0.0; 0.02)
Pain (/10)^‡^	0 (0–0)		1 (0–2)		0 (0–2.25)		0 (0–0)		*p* = 0.023*	0.10 (0.08; 0.11)
Swelling (mm)^†^	53.93	2.88	54.59	3.79	55.38	5.07	53.58	3.68	*p* = 0.57	0.03 (0; 0.11)
WBLT (cm)^‡^	14.06	2.80	12.41	3.72	13.41	3.72	12.29	2.82	*p* = 0.27	0.01 (0; 0.03)
Arthrokinematic (deg)^‡^	15.20	5.07	17.77	5.47	15.07	6.07	14.91	5.65	*p* = 0.31	0.01 (0; 0.02)
mSEBT (% leg length)^†^	87.60	4.74	85.95	5.24	85.18	6.15	85.95	6.20	*p* = 0.55	0.03 (0; 0.11)
mSEBT_Anterior^†^	90.70	4.72	87.51	5.24	90.46	5.30	89.89	5.15	*p* = 0.22	0.06 (0; 0.17)
mSEBT_Postero-medial^†^	84.74	7.01	84.16	6.31	81.90	9.51	84.50	8.68	*p* = 0.7	0.02 (0; 0.09
mSEBT_Postero-lateral^†^	87.42	6.54	85.78	7.51	83.82	6.98	83.55	8.01	*p* = 0.32	0.05 (0; 0.15)
BESS_OE (N errors)^‡^	0 (0–1.25)		0 (0–2)		0 (0–1)		0 (0–1)		*p* = 0.79	<0.001 (undefined)
BESS_CE (N errors)^‡^	10 (10–10)		10 (10–10)		10 (10–10)		10 (10–10)		*p* = 0.88	<0.001 (undefined)

BESS_EC, balance error scoring system, eyes closed; BESS_EO, BESS, eyes open; BMI, body mass index; CAI, chronic ankle instability; CAIT, cumberland ankle instability tool; FAAM_ADL, foot and ankle ability measure, activities of daily life subscale; FAAM_Sport, FAAM, sport subscale; LAS, lateral ankle sprain; M/F, male/female ratio; mSEBT, modified star excursion balance test; n, number of participants; SD, standard deviation; WBLT, weight bearing lunge test; ^†^(parametric tests were performed), ^‡^(non-parametric tests were performed), *and bold (significant at *p* < 0.05).

A total of 150 people completed the recruitment process and 70 were enrolled in the study. They were categorized as healthy (*n* = 24), coper (*n* = 13), LAS (*n* = 17), and CAI (*n* = 16). Weight was the only demographic variable that was normally distributed. Gender distribution, age, weight, height, BMI were similar between groups, allowing comparison.

### 3.2 Clinical assessment

Only swelling and the four outcomes related to mSEBT were normally distributed. Participants with CAI reported significantly more perceived instability according to CAIT and lower self-reported function according to both FAAM subscales compared to healthy controls and copers (Table 1; Table 3). Similarly, people with a recent LAS also scored lower on CAIT and FAAM questionnaires compared to healthy controls and coper individuals. No between-group difference was identified for Tegner level of physical activity and pain.

All outcomes of the physical examination were not significantly different between groups.

### 3.3 Walking biomechanics

Three participants of the LAS group were removed from the analyses, because the values of all variables were extremely low (around zero), due to technical issues during the recording.

**Figure 4:**
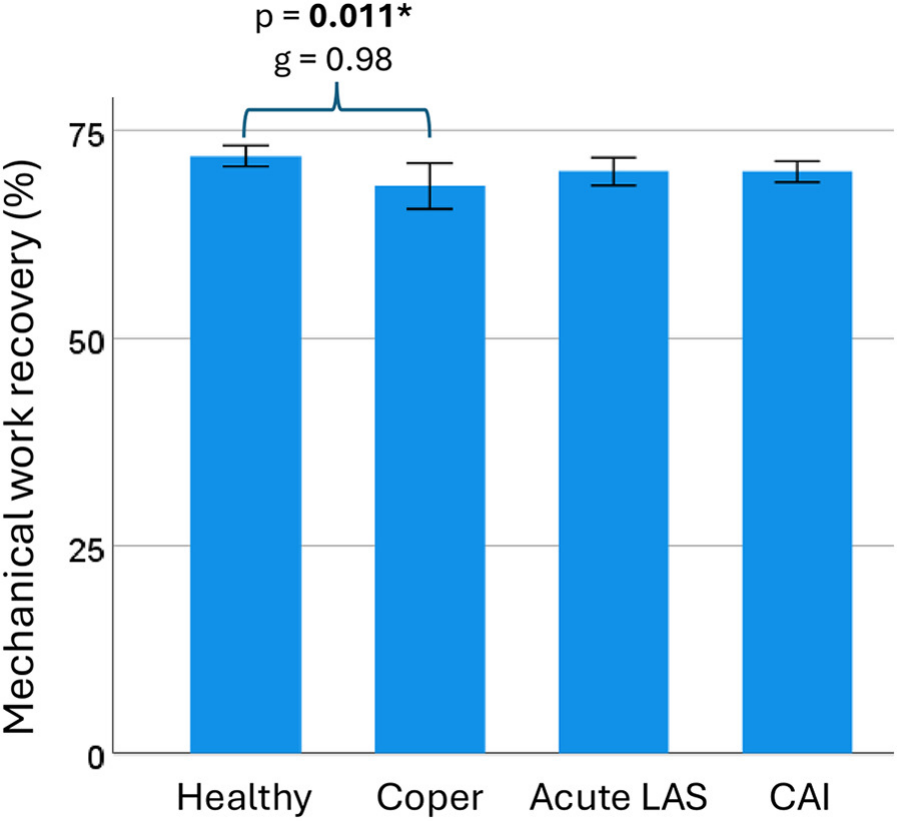
Mechanical work recovery during walking. Mechanical work recovery during walking was statistically higher in the healthy control group compared to the coper group. CAI, chronic ankle instability; LAS, lateral ankle sprain; g, Hedge's g effect size; p, *p*-value. * and bold (statistically significant at *p* < 0.05). Error bar: 95% confidence interval of the mean.

Walking biomechanical outcomes are available in Table 2. Participants walked at a mean speed of 1.29 m/s in copers and 1.31 m/s in the other groups (*p* = 0.96). Healthy controls showed a significantly higher mechanical work recovery compared to the coper group [t(35) = 2.9, *p* = 0.011, g = 0.98, 95% CI = 0.27–1.67] (Table 3; Figure 4). There were no significant differences for all other outcomes.

**Table 2:** Walking biomechanics.

Outcomes	Healthy controls (*n* = 24)		Recent LAS (*n* = 14)		CAI (*n* = 16)		Copers (*n* = 13)		*p*-value	*η*² effect size (95% CI)
	Mean	SD	Mean	SD	Mean	SD	Mean	SD		
Walking speed (m/s)^†^	1.31	0.09	1.31	0.15	1.31	0.09	1.29	0.07	*p* = 0.96	0.005 (0; 0.02)
Base of support width (cm)^†^	11.83	2.04	12.32	2.92	12.13	2.69	11.69	1.40	*p* = 0.88	0.01 (0; 0.05)
Braking Peak Force (%BW)^†^	17.84	2.14	17.96	2.83	17.64	3.12	17.25	2.28	*p* = 0.89	0.01 (0; 0.05)
COM Path (mm)^‡^	95.91	12.62	102.40	11.73	99.46	18.97	95.88	10.16	*p* = 0.53	<0.001 (undefined)
COM Vertical Displ. (mm)^‡^	37.33	6.10	39.14	6.00	38.69	8.80	37.03	4.42	*p* = 0.85	<0.001 (undefined)
Contact Duration (%stride)^†^	63.24	0.78	63.45	0.82	63.50	0.95	63.27	0.78	*p* = 0.74	0.02 (0; 0.08)
Double support duration (%stride)^†^	13.34	0.79	13.38	0.87	13.46	0.99	13.45	0.80	*p* = 0.97	0.004 (0; 0.01)
Duty Factor^‡^	0.63	0.01	0.63	0.01	0.64	0.01	0.63	0.01	*p* = 0.53	<0.001 (undefined)
Loading peak force (%BW)^‡^	115.16	6.98	115.91	8.12	112.36	9.37	115.63	7.18	*p* = 0.23	0.02 (0.01; 0.04)
Loading Rate (%BW/ms)^†^	1.01	0.21	0.89	0.14	0.92	0.15	0.87	0.15	*p* = 0.09	0.10 (0; 0.22)
Propulsive Peak Force (%BW)^‡^	20.13	1.61	21.38	2.84	20.54	3.13	20.88	2.38	*p* = 0.19	0.03 (0.01; 0.05)
Push-Off Rate (%BW/ms)^‡^	1.19	0.11	1.21	0.19	1.20	0.12	1.24	0.11	*p* = 0.35	0.004 (0; 0.02)
Single Support Duration (%stride)^†^	36.69	0.85	36.59	1.11	36.59	1.23	36.42	0.90	*p* = 0.9	0.009 (0; 0.04)
Step Length (cm)^†^	70.04	4.12	71.07	5.15	70.83	5.84	68.42	4.41	*p* = 0.48	0.04 (0; 0.13)
Vertical Impulse (%BW.s)^‡^	54.48	2.56	55.41	4.10	55.15	2.72	54.14	2.86	*p* = 0.85	<0.001 (undefined)
Cadence (spm)^†^	110.23	5.08	108.97	8.27	109.18	5.32	110.85	5.03	*p* = 0.81	0.02 (0; 0.07)
Mechanical work Fore-aft. (J/kg.m)^†^	0.45	0.04	0.47	0.06	0.45	0.06	0.46	0.06	*p* = 0.63	0.03 (0; 0.10)
Mechanical work vertical (J/kg.m)^†^	0.51	0.07	0.54	0.07	0.53	0.08	0.53	0.05	*p* = 0.57	0.03 (0; 0.11)
Mechanical work recovery (%)^†^	71.91	3.00	70.05	2.94	70.04	2.40	68.31	4.56	*p* = 0.016*	0.15 (0.01; 0.28)
Stride Duration (ms)^†^	1,090.85	49.68	1,107.41	87.64	1,101.70	55.38	1,084.80	49.82	*p* = 0.74	0.02 (0; 0.08)

BW, body weight; CAI, chronic ankle instability; LAS, lateral ankle sprain; SD, standard deviation; spm, steps per minute; ^†^(parametric tests were performed), ^‡^(non-parametric tests were performed), *and bold (significant at *p* < 0.05).

**Table 3:** *Post-hoc* tests.

Outcomes	Healthy controls vs. copers	Healthy control vs. acute LAS	Healthy control vs. CAI	Coper vs. acute LAS	Coper vs. CAI	Acute LAS vs. CAI
CAIT (/30)^‡^	*p* = 1, *r* = 0.14	*p* < 0.001*, *r* = 0.73	*p* < 0.001*, *r* = 0.86	*p* = 0.001*, *r* = 0.68	*p* **<** 0.001*, *r* = 0.85	*p* = 1, *r* = 0.02
FAAM_ADL (%)^‡^	*p* = 1, *r* = 0.08	*p* = 0.016*, *r* = 0.47	*p* < 0.001*, *r* = 0.64	*p* = 0.042*, *r* = 0.49	*p* < 0.001*, *r* = 0.67	*p* = 1, *r* = 0.05
FAAM_Sport (%)^‡^	*p* = 1, *r* = 0.02	*p* = <0.001*, *r* = 0.62	*p* < 0.001*, *r* = 0.64	*p* = 0.006*, *r* = 0.60	*p* = 0.004*, *r* = 0.63	*p* = 1, *r* = 0.20
Pain (/10)^‡^	*p* = 1, *r* = 0.04	*p* = 0.0501, *r* = 0.41	*p* = 0.18, *r* = 0.34	*p* = 0.31, *r* = 0.35	*p* = 0.10, *r* = 0.30	*p* = 1, *r* = 0.05
Mechanical work recovery (%)^†^	*p* = 0.011*, g = 0.98, CI = [0.27; 1.67]	*p* = 0.55, g = 0.61, CI = [−0.05; 1.27]	*p* = 0.46, g = 0.66, CI = [0.02; 1.30]	*p* = 0.99, g = −0.45, CI = [−1.18; 0.30]	*p* = 0.94, g = −0.48, CI = [−1.19; 0.25]	*p* = 1, g = −0.01, CI = [−0.70; 0.69]

CAI, chronic ankle instability; CAIT, cumberland ankle instability tool; CI, 95% confidence interval on the effect size; FAAM_ADL, foot and ankle ability measure, activities of daily life subscale; FAAM_Sport, FAAM, sport subscale; LAS, lateral ankle sprain; g, Hedge's g effect size related to a *post-hoc t*-test; r, effect size related to a Mann–Whitney *post-hoc* test; ^†^(parametric tests were performed), ^‡^(non-parametric tests were performed), *and bold (significant at *p* < 0.05, *p*-values were corrected to account for multiple comparisons).

### 3.4 Running biomechanics

Running biomechanical outcomes are available in Table 4. Results were not available for one participant with CAI and one participant with LAS because their data were not correctly saved.

**Table 4:** Running biomechanics.

Outcomes	Healthy controls (*n* = 24)		Recent LAS (*n* = 16)		CAI (*n* = 15)		Copers (*n* = 13)		*p*-value	*η*² effect size
	Mean	SD	Mean	SD	Mean	SD	Mean	SD		
Running speed (m/s)^†^	2.72	0.31	2.75	0.35	2.72	0.45	2.69	0.28	*p* = 0.98	0.003 (0; 0)
Base of support width (cm)^†^	6.71	2.94	6.97	2.37	6.86	2.21	6.31	1.82	*p* = 0.90	0.01 (0; 0.04)
Braking Peak Force (%BW)^†^	27.83	4.26	26.93	3.93	26.45	4.79	26.74	4.17	*p* = 0.77	0.02 (0; 0.08)
COM Path (mm)^†^	169.94	20.80	178.22	23.00	170.98	27.10	172.33	22.68	*p* = 0.72	0.02 (0; 0.08)
COM Vertical Displ. (mm)^†^	83.66	10.21	86.91	11.92	83.31	13.99	84.43	11.62	*p* = 0.82	0.01 (0; 0.07)
Contact Duration (%stride)^‡^	38.99	2.91	38.20	2.63	39.01	4.20	38.61	3.39	*p* = 0.50	<0.001 (undefined)
Aerial duration (%stide)^‡^	11.06	3.15	11.80	2.54	10.90	4.27	11.34	3.49	*p* = 0.53	<0.001 (undefined)
Duty Factor^‡^	0.39	0.03	0.38	0.03	0.39	0.04	0.39	0.03	*p* = 0.50	<0.001 (undefined)
Impact Peak Force (%BW)^‡^	127.75	52.47	141.98	45.74	148.15	21.43	144.52	22.75	*p* = 0.92	<0.001 (undefined)
Leg Stiffness (%BW/m)^‡^	1,431.31	137.60	1,524.84	347.00	1,438.59	241.61	1,577.43	505.15	*p* = 0.82	<0.001 (undefined)
Loading Rate (%BW/ms)^‡^	4.46	1.12	4.88	1.58	5.11	1.17	4.22	1.29	*p* = 0.17	0.03 (0.02; 0.06)
Propulsive Peak Force (%BW)^†^	21.82	3.69	21.54	3.42	20.99	4.99	21.61	3.17	*p* = 0.93	0.01 (0; 0.03)
Step Length (cm)^†^	101.50	11.68	102.40	13.23	100.73	14.70	100.31	10.69	*p* = 0.97	0.004 (0; 0.005)
Cadence (spm)^‡^	162.76	6.47	159.86	7.79	160.12	8.71	160.65	4.75	*p* = 0.60	<0.001 (undefined)
Mechanical work Fore-aft (J/kg.m)^‡^	0.49	0.05	0.47	0.06	0.47	0.06	0.49	0.04	*p* = 0.93	<0.001 (undefined)
Mechanical work Total (J/kg.m)^†^	1.27	0.10	1.30	0.10	1.27	0.09	1.29	0.10	*p* = 0.74	0.02 (0; 0.08)
Mechanical work vertical (J/kg.m)^†^	0.80	0.09	0.84	0.10	0.82	0.12	0.83	0.09	*p* = 0.56	0.03 (0; 0.11)
Stride duration (ms)^†^	738.47	28.74	752.44	37.23	751.67	40.44	747.67	21.53	*p* = 0.50	0.04 (0; 0.12)

BW, body weight; CAI, chronic ankle instability; LAS, lateral ankle sprain; SD, standard deviation; spm, steps per minute; ^†^(parametric tests were performed), ^‡^(non-parametric tests were performed). Significance was set to *p* < 0.05

Participants ran at a mean speed that ranged from 2.69 m/s in copers to 2.75 m/s in recent LAS (*p* = 0.98). None of the analyzed biomechanical variables statistically differed between groups.

## 4 Discussion

This study investigated the biomechanical differences in walking and running among runners categorized as CAI, copers, those with acute LAS and control subjects. We identified no major differences between groups during running, and a higher mechanical work recovery during walking in healthy controls compared to copers. These results suggest a limited influence of CAI and LAS on running biomechanics under controlled conditions. To the best of our knowledge, this is the first study to compare running biomechanics among the four clinical categories related to ankle sprains. Comparing copers to individuals experiencing acute LAS and CAI provides valuable insights into the contributing factors and unique aspects of the rehabilitation process, as all these participants have faced a similar initial injury (7).

Previous studies have reported mixed results concerning the impact of CAI on walking and running biomechanics. On the one hand, studies that specifically focused on runners have found evidence of altered kinetics compared to their healthy counterparts (38, 39). For instance, college-aged runners with CAI exhibited higher impact peak forces during running at a speed of 3.3 m.s^−1^ (38). Additionally, young adult recreational runners with CAI exhibited increased impact decelerations and longer foot contact times when running at a self-selected comfortable speed (39), and female runners classified as copers exhibited increased vertical ground reaction forces compared to healthy controls while walking (11). On the other hand, several other studies have reported no significant differences in spatiotemporal parameters and ground reaction forces between CAI and healthy controls, nor between CAI and copers. However, these studies did not specifically investigate runners (9, 10, 40–42). Similarly, we objectified no between-group differences during running, despite notable clinical symptoms. This suggests that running at a controlled speed remains highly accessible for individuals with LAS or CAI, and may provide opportunities to adjust the rehabilitation timeline. In line with this, Rhon et al. (43) reported that early rehabilitation can reduce the recurrence rate and financial burden associated with LAS rehabilitation. We recommend that clinicians consider implementing an early running protocol for runners with LAS when clinically appropriate. In contrast, copers exhibited a decreased mechanical work recovery during walking, indicating an impaired ability to convert potential and kinetic energies (44, 45). Although the comparisons between controls vs. CAI and acute LAS groups did not reach statistical significance, the corresponding effect sizes indicated similar trends (Table 3). Our results thus suggest a general reduction in walking efficiency among individuals with a history of ankle sprain. The FAAM scores reflected heterogeneous levels of perceived function in the CAI and acute LAS groups, which may help explain why reductions in mechanical work recovery were not statistically consistent. Still, subtle alterations in distal or proximal joint kinematics are frequently reported following LAS and among individuals with CAI (8, 46), which may impair this energetic exchange. Moreover, coordination patterns in copers and CAI differ from healthy controls during dynamic tasks, supporting the presence of altered and potentially compensatory control strategies (47). Since mechanical work recovery is specific to walking and not captured during running, it may serve as a more sensitive indicator of gait impairments. We encourage further investigation into its clinical and research relevance. Taken together, these findings underscore that standard biomechanical assessments under controlled conditions may fail to detect subtle, yet meaningful, functional impairments in individuals with CAI.

This study is the first to systematically assess ROAST outcomes among runners with different clinical trajectories following an initial LAS, providing clinically relevant information for tailored rehabilitation strategies (15). Among the questionnaires of the anamnesis, we found more perceived ankle instability and worse self-reported function in CAI compared to healthy, in CAI compared to copers, in recent LAS compared to healthy, and in recent LAS compared to copers. These results highlight the importance of considering perceived instability and self-reported function in the assessment of LAS and CAI. The implications of these results are twofold. First, our study demonstrates that some individuals with a recent LAS are characterized by important levels of perceived instability and low self-reported function. Second, despite these sensory-perceptual alterations, we identified no major between-group differences in the three motor-behavioral outcomes assessed: walking and running biomechanics, static and dynamic balance, and physical activity. Notably, balance performance did not differ significantly between groups, despite its established role as a risk factor for recurrent LAS (16). In terms of static balance, all participants made very few errors with eyes open and many errors with eyes closed during the BESS, suggesting possible ceiling and floor effects, respectively. Other relevant sensory-perceptual factors that influence clinical outcome after a LAS include ankle proprioception and kinesiophobia (5). Although not assessed in the present study, recent work has shown that ankle inversion proprioception measured during dynamic tasks is significantly associated with CAIT scores (48, 49). These findings highlight that alterations in sensory-perceptual processing may be more prominent in CAI and LAS than global motor-behavioral deficits detectable under steady-state testing conditions. Lastly, although ankle strength was not assessed in this study, prior research has reported strength deficits in individuals with CAI, which may also contribute to recurrent LAS (16). Whether such impairments are present in recreational runners with regular sport participation remains to be determined.

Pain is another important sensory-perceptual factor that is part of the ROAST (5, 15). In our study, pain levels were relatively low. Still, prior research has shown that pain is common in CAI populations (50), and that its severity contributes substantially to self-reported function and disability (51). Although our study was not designed to isolate the effects of pain on gait, recent evidence suggests that chronic pain can alter lower-limb motor strategies during sport-specific tasks and influence postural control (52, 53). These findings support the need for future studies to examine how pain may affect gait and running biomechanics among individuals with CAI.

Several factors may explain the differences in findings across studies. The external devices used by runners can influence running biomechanics (54). In several studies, participants were instructed to run barefoot (11, 41), in customized shoes (40), or on a treadmill (10, 38). Furthermore, some studies define the control group inconsistently, not differentiating between copers and healthy control participants (41). The definition of perceived instability also exhibits considerable heterogeneity—particularly regarding the cutoff scores used in the questionnaires (5, 22, 55)—which complicates comparisons across studies. Our findings suggest that standard biomechanical assessments conducted while running at a self-selected comfortable speed may not be the most effective method for detecting functional impairments in CAI populations. Future studies should investigate running biomechanics at higher speeds. Moreover, since ankle sprains are more likely to occur during directional changes (12), side-cutting and reactive stabilization exercises may be more suitable for identifying deficits in movement patterns. The frequent lack of significant biomechanical differences observed during walking and running, as reported in previous studies and in our current research, raises important questions about the methods used to assess movement patterns in individuals with CAI. Due to the limitations of standard gait analysis, advanced biomechanical modeling techniques have been developed. Researchers commonly assess joint kinematics and kinetics by placing reflexive markers on the lower limbs (56, 57). Research utilizing models that segment the foot into multiple parts has revealed that individuals with CAI exhibit altered rearfoot motion compared to healthy participants (8, 58, 59), as well as altered forefoot kinematics (58). Recent advancements now enable the characterization of ankle and foot joint loading patterns by integrating kinematic data with foot pressure measurements (60). Additionally, ligament strains and musculotendon actuators can be incorporated into multi-body models for a more comprehensive assessment of the ankle joint (61, 62). Future studies should adopt these musculoskeletal modeling techniques to provide deeper insight into how joint and ligament loading vary between individuals with and without CAI.

Some limitations need to be acknowledged. Firstly, although it is certain that participants in the acute LAS group sustained their last ankle sprain less than one year ago, the precise duration since that last injury is unknown. Additionally, recall bias influences ankle-sprain research, as group classification relies on self-reported injury history (6). Other confounding factors include the varying levels of physical abilities and the footwear used by the participants. Secondly, while copers were not specifically controlled for return to pre-injury level, their inclusion provides a valuable first step in understanding why some individuals recover fully while others develop CAI. Thirdly, this study included a convenience sample of runners without a predefined sample size calculation, meaning statistical power may be inadequate. Besides, a cross-sectional design is not adapted to infer causal interpretations. The external validity of our findings apply to young recreationally active runners walking and running at self-selected comfortable speeds. Further research should confirm these findings for walking and running at higher speeds. Finally, although gait parameters provide valuable insights, they may not fully capture the multi-factorial nature of motor-behavioral impairments in CAI. Our findings align with the updated model proposed by Hertel & Corbett (5), indicating that the neurosignature of individuals with CAI arises from a cumulative range of subtle to mild impairments. Future research should integrate multi-joint kinematic modeling and neuromuscular assessments to provide a more comprehensive understanding of movement dysfunction in CAI.

## 5 Conclusion

In summary, this study found no differences between runners with and without CAI during running at comfortable speed. However, copers exhibited reduced walking efficiency, suggesting potential lingering impairments. Clinicians should carefully assess sensory-perceptual impairments, including perceived instability and self-reported function. Future research should examine more challenging tasks, such as running at higher speeds and directional changes, while incorporating advanced biomechanical modeling to better understand joint loading and injury mechanisms.

## Data Availability

The raw data supporting the conclusions of this article will be made available by the authors, without undue reservation.
