# Supplementary material for: Ankle sprain history and clinical outcome have limited influence on walking and running biomechanics among runners: a cross-sectional study
Source: Front Sports Act Living. 2025 Sep 8;7:1553995. doi: 10.3389/fspor.2025.1553995 (PMC12452186; doi:10.3389/fspor.2025.1553995)
Supplement: Supplementary file 1 [file Datasheet1.docx]

**Definitions of the walking and running biomechanical variables**

The descriptions of the biomechanical variables are based on the **user manual** provided by **h/p/cosmos® (Arsalis)**. The user manual was retrieved online from <https://www.arsalis.com/wp-content/uploads/2023/12/User-manual_hpcosmos_gaitway_3d.pdf>, accessed February, 14, 2025.

**Aerial duration (%stride)**: Time during which no force is applied during a step, which corresponds to no foot in contact with the ground.
*This outcome was only reported for running.*

**Base of support width (cm)**: Average lateral distance between both feet during the contact phases.

**Braking peak force (%BW)**: Fore-afterwards braking peak force decelerating the body.

**Cadence (spm)**: The number of foot strikes per minute.

**Center of mass path (mm)**: Length of the three-dimensional path traveled by the body center of mass during a step.

**Center of mass vertical displacement (mm)**: Vertical displacement of the body center of mass during a step, obtained by double integration of the vertical force signal.

**Contact duration (%stride)**: Duration for which force is applied during a foot strike, which corresponds to the duration from heel contact to toe-off.

**Double support duration (%stride)**: Duration of the body supported by both feet.
*This outcome was only reported for walking.*

**Duty factor**: The ratio of contact duration to stride duration.

**Impact Peak Force (%BW)**: First vertical peak on the vertical force curve shortly after foot impact. *This outcome was only reported for running.*

**Leg stiffness (%BW/m)**: Stiffness of the leg spring computed as the ratio of the maximal vertical force to the compression of the leg.
*This outcome was only reported for running.*

**Loading peak force (%BW)**: First vertical peak on the vertical force curve, shortly after heel contact.
*This outcome was only reported for walking.*

**Loading Rate (%BW/ms)**: Slope of the vertical force time curve during the loading phase, taken from one point at 20% of first peak force and one point at 80% of first peak force.

**Mechanical work fore-afterwards (J/kg.m)**: Fore-afterwards positive mechanical work to accelerate the body center of mass in the forward direction. This outcomes is obtained by summing the increments of the kinetic fore-afterwards mechanical energy curve during the stride. The value is then normalized per body mass and distance.

**Mechanical work vertical (J/kg.m)**: Vertical positive mechanical work to lift and accelerate the body center of mass in the upward direction. This outcomes is computed by summing the increments of the vertical mechanical energy curve during the stride. The value is then normalized per body mass and distance.

**Mechanical work recovery (%)**: Percentage of energy recovered during locomotion due to the exchange of energy between potential and kinetic energies.

**Propulsive peak force (%BW)**: Fore-afterwards propulsive peak force accelerating the body.

**Push-off Rate (%BW/ms)**: Slope of the vertical force time curve during the unloading phase, taken from one point at 80% of active peak force and one point at 20% of active peak force.
*This outcome was only reported for walking.*

**Single support duration (%stride)**: The period during a foot strike in which the body is supported by only one foot.
*This outcome was only reported for walking.*

**Step length (cm)**: The fore-afterwards distance from the initial contact of a foot to the initial contact of the contralateral foot.

**Stride duration (ms)**: The time duration from the initial contact of the left foot to the next initial contact of the left foot.

**Vertical impulse (%BW.s)**: The integral, or area under the vertical force time curve during the foot strike.
