# Supplementary material for: Ankle sprain history and clinical outcome have limited influence on walking and running biomechanics among runners: a cross-sectional study
Source: Front Sports Act Living. 2025 Sep 8;7:1553995. doi: 10.3389/fspor.2025.1553995 (PMC12452186; doi:10.3389/fspor.2025.1553995)
Supplement: Supplementary file 2 [file Datasheet2.docx]

STROBE Statement—Checklist of items that should be included in reports of ***cross-sectional studies***

|  | Item No | | Recommendation | Section |
| --- | --- | --- | --- | --- |
| **Title and abstract** | 1 | | (*a*) Indicate the study’s design with a commonly used term in the title or the abstract | Title + Abstract |
|  |  |  | (*b*) Provide in the abstract an informative and balanced summary of what was done and what was found | Abstract |
| Introduction | |  |  |  |
| Background / rationale | 2 | | Explain the scientific background and rationale for the investigation being reported | Introduction |
| Objectives | 3 | | State specific objectives, including any prespecified hypotheses | Introduction (last paragraph) |
| Methods | |  |  |  |
| Study design | 4 | | Present key elements of study design early in the paper | “Study design” |
| Setting | 5 | | Describe the setting, locations, and relevant dates, including periods of recruitment, exposure, follow-up, and data collection | “Testing procedure” |
| Participants | 6 | | (*a*) Give the eligibility criteria, and the sources and methods of selection of participants | “Participants” |
| Variables | 7 | | Clearly define all outcomes, exposures, predictors, potential confounders, and effect modifiers. Give diagnostic criteria, if applicable | “Testing procedure” |
| Data sources/ measurement | 8 | | For each variable of interest, give sources of data and details of methods of assessment (measurement). Describe comparability of assessment methods if there is more than one group | “Testing procedure” |
| Bias | 9 | | Describe any efforts to address potential sources of bias | *Recall bias recognized in limitations* |
| Study size | 10 | | Explain how the study size was arrived at | *Lack of sample size calculation recognized in limitations* |
| Quantitative variables | 11 | | Explain how quantitative variables were handled in the analyses. If applicable, describe which groupings were chosen and why | “Statistical analyses”, first paragraph |
| Statistical methods | 12 | | (*a*) Describe all statistical methods, including those used to control for confounding | “Statistical analyses”, second paragraph |
|  |  |  | (*b*) Describe any methods used to examine subgroups and interactions | / |
|  |  |  | (*c*) Explain how missing data were addressed | *Results: Missing data not included in the analyses.* |
|  |  |  | (*d*) If applicable, describe analytical methods taking account of sampling strategy | / |
|  |  |  | (*e*) Describe any sensitivity analyses | / |
| Results | |  |  |  |
| Participants | 13 | | (a) Report numbers of individuals at each stage of study—eg numbers potentially eligible, examined for eligibility, confirmed eligible, included in the study, completing follow-up, and analysed | “Participants” |
|  |  |  | (b) Give reasons for non-participation at each stage | *All enrolled participants completed the study, since there was only one lab-visit.* |
|  |  |  | (c) Consider use of a flow diagram | / |
| Descriptive data | 14 | | (a) Give characteristics of study participants (eg demographic, clinical, social) and information on exposures and potential confounders | “Participants” |
|  |  |  | (b) Indicate number of participants with missing data for each variable of interest | “Walking biomechanics” and “Running biomechanics”: *Missing data are reported.* |
| Outcome data | 15 | | Report numbers of outcome events or summary measures | / |
| Main results | 16 | | (*a*) Give unadjusted estimates and, if applicable, confounder-adjusted estimates and their precision (eg, 95% confidence interval). Make clear which confounders were adjusted for and why they were included | “Clinical assessment” to “Running biomechanics”: *Main results are reported.*  Tables: *Effect size estimates* *and 95% confidence intervals are reported.* |
|  |  |  | (*b*) Report category boundaries when continuous variables were categorized | / |
|  |  |  | (*c*) If relevant, consider translating estimates of relative risk into absolute risk for a meaningful time period | / |
| Other analyses | 17 | | Report other analyses done—eg analyses of subgroups and interactions, and sensitivity analyses | / |
| Discussio | |  |  |  |
| Key results | 18 | | Summarise key results with reference to study objectives | First paragraph of discussion |
| Limitations | 19 | | Discuss limitations of the study, taking into account sources of potential bias or imprecision. Discuss both direction and magnitude of any potential bias | Last paragraph of discussion |
| Interpretation | 20 | | Give a cautious overall interpretation of results considering objectives, limitations, multiplicity of analyses, results from similar studies, and other relevant evidence | “Conclusion” |
| Generalisability | 21 | | Discuss the generalisability (external validity) of the study results | Last paragraph of discussion: *“young recreationally active runners …”* |
| Other information | |  |  |  |
| Funding | 22 | | Give the source of funding and the role of the funders for the present study and, if applicable, for the original study on which the present article is based | “Funding” |

Strobe guidelines for Strengthening the Reporting of Observational Studies in Epidemiology (von Elm et al., 2008) can be retrieved from Equator_network website.

Bibliography

Equator_network. *The Strengthening the Reporting of Observational Studies in Epidemiology (STROBE) Statement: guidelines for reporting observational studies*. Retrieved 16 March, 2025 from <https://www.equator-network.org/reporting-guidelines/strobe/>

von Elm, E., Altman, D. G., Egger, M., Pocock, S. J., Gøtzsche, P. C., & Vandenbroucke, J. P. (2008, Apr). The Strengthening the Reporting of Observational Studies in Epidemiology (STROBE) statement: guidelines for reporting observational studies. *J Clin Epidemiol, 61*(4), 344-349. <https://doi.org/10.1016/j.jclinepi.2007.11.008>
